# First Use of Upfront Polatuzumab Vedotin in Post-transplant Lymphoproliferative Disorder: A Case Report

**DOI:** 10.7759/cureus.56409

**Published:** 2024-03-18

**Authors:** Sreshta Paranji, Amir Steinberg

**Affiliations:** 1 Internal Medicine, Westchester Medical Center, Valhalla, USA; 2 Hematology and Oncology, Westchester Medical Center, Valhalla, USA

**Keywords:** r-chop therapy, large b-cell lymphoma, lymphoproliferative malignancy, post-transplant lymphoproliferative disorders, polatuzumab vedotin

## Abstract

Post-transplant lymphoproliferative disorder (PTLD) is a condition that is highly variable in presentation but life-threatening for post-transplant, immunosuppressed patients. Current standard management in PTLD sees the use of a chemoimmunotherapy regimen similar to the management of diffuse large B-cell lymphoma. Here, we discuss the case of a 33-year-old male with a history of renal transplant, hemodynamically stable, who presented with fevers and night sweats lasting one month. Investigations revealed multiple masses in his liver, the largest of which was biopsied and revealed diffuse large B-cell lymphoma. PTLD is an important malignancy in patients who have received immunosuppression, but the treatment is heterogeneous, based on subtype and patient status. This case, where the addition of polatuzumab to the standard rituximab, cyclophosphamide, doxorubicin, and vincristine (R-CHOP) regimen led to favorable results, demonstrates the potential for a new standard treatment regimen for this disease.

## Introduction

Post-transplant lymphoproliferative disorder (PTLD) is overall rare, but it is the most common malignancy after a solid organ transplant. Risk factors for developing PTLD include the Epstein-Barr virus (EBV) status of the recipient as well as the degree of immunosuppression [1]. Patients often present with fevers, weight loss, and fatigue, among other non-specific symptoms. In terms of therapy, current standard management of PTLD refractory to immunosuppression reduction (RIS) incorporates the use of chemoimmunotherapy, typically the rituximab, cyclophosphamide, doxorubicin, and vincristine (R-CHOP) regimen, similar to the management of diffuse large B-cell lymphoma (DLBCL) [2]. Recently, the use of polatuzumab vedotin, the antibody-drug conjugate targeting the cluster of differentiation marker 79 (CD79), was approved by the FDA for the treatment of DLBCL in patients with an International Prognostic Index (IPI) score of 2 or higher [2]. Given the improved progression-free survival of Pola-R-CHP (polatuzumab and R-CHOP combined regimen) compared to R-CHOP in DLBCL (76.7% vs 70.2%) [3], we elected to give the newly approved combination for PTLD. Additionally, studies show that EBV with PTLD tends to more often be associated with the non-germinal center B cell (non-GCB) subtype which, in subset analysis, benefits even further from Pola-R-CHP [4] as this subtype shows an 83.9% vs 58.5% progression-free survival. The preliminary findings of this article were previously presented as a meeting abstract at the 2023 SOHO Annual Meeting on September 6, 2023.

## Case presentation

**Figure 1:**
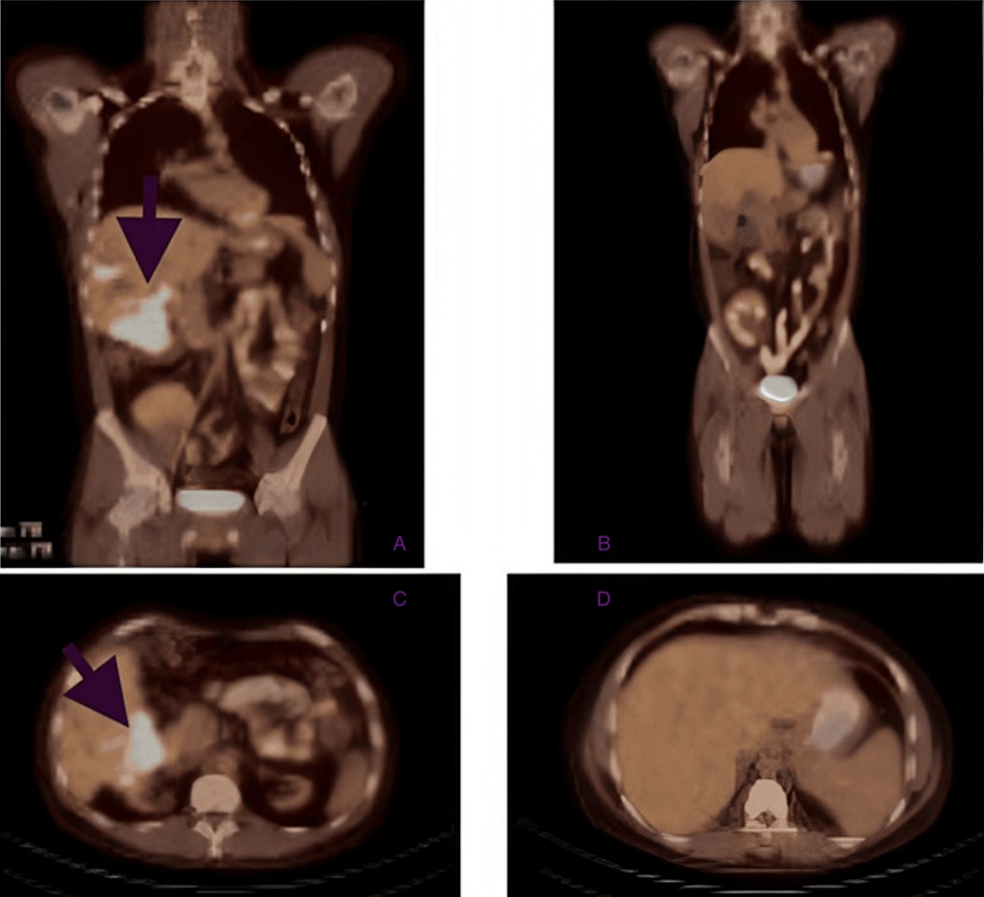
Before and after two cycles PET-CT scans from May 23, 2023 (A and C), after four weekly doses of rituximab. The top left (A) view is a coronal cut including the largest hepatic lesion visualized (arrow), and the bottom left (C) shows the transverse cut (arrow). This is juxtaposed to PET-CT scans from July 10, 2023 (B and D) after cycle 2 including the Pola-R-CHP regimen, showing no clear evidence of lymphoma in the liver. The top coronal cut (B) shows improvement, and the transverse cut (D) shows evidence of remission of the disease. PET-CT: positron emission tomography-computer tomography, Pola-R-CHP: polatuzumab and R-CHOP combined regimen.

A 33-year-old male with a past medical history of immune thrombocytopenia and end-stage renal disease due to IgA nephropathy presented with intermittent fevers and night sweats for one month, two years post-kidney transplant. The patient had been on belatacept for immunosuppression after his transplant. At the time of presentation, his EBV polymerase chain reaction (PCR) level was elevated at 2.5 million IU/mL with a lactate dehydrogenase (LDH) level of 1,732 U/L. His workup included a computed tomography (CT) scan which showed multiple masses in the right (R) lobe of his liver, the largest of which measured 12.7 cm. The ensuing liver biopsy showed a diagnosis of diffuse large B-cell lymphoma, of the non-GCB immunophenotype. His Ki-67, a nuclear protein involved in cell proliferation which can be used to evaluate the aggressiveness of lymphoma, was 80%-90%. His Fluorescence in situ Hybridization (FISH) was negative for markers B-cell lymphoma 2, B-cell lymphoma 6, and MYC gene, which assisted in the diagnosis of PTLD. His IPI was 2 due to his staging being stage 4 from liver involvement and the aforementioned elevated LDH. The patient was given four weekly doses of rituximab, and his follow-up positron emission tomography-computer tomography (PET-CT) scan showed an overall improvement with, most notably, a reduction in liver lesion size. The LDH had also improved, though still elevated. His EBV-PCR level was decreased. However, given that the disease was still present, in an effort to push the patient into remission, we chose Pola-R-CHOP rather than RCHOP or dose-adjusted etoposide, prednisone, vincristine, cyclophosphamide, doxorubicin, rituximab (DA-EPOCH-R). After two cycles of the regimen, the patient’s PET-CT scans showed no clear evidence of lymphoma in the liver (Figures 1A-1D). After completing six cycles of this regimen, the patient’s EBV was undetectable.

**Figure 2:**
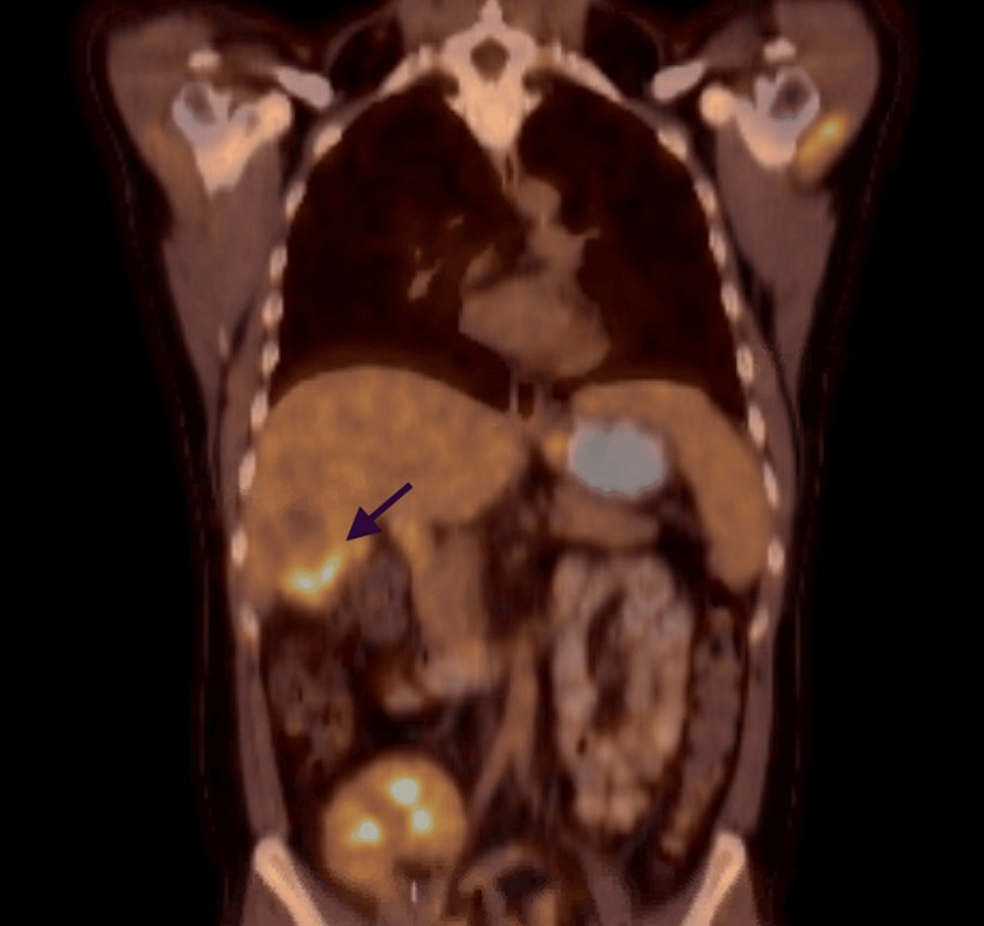
After six cycles of the regimen, a new area of focus Arrow shows the new area of uptake that was concerning.

In the patient’s first surveillance PET-CT scan after he received his sixth cycle, an interval increase in uptake was noted in the R inferior hepatic lobe, concerning metastatic disease (Figure 2). The lesion was 9.3×5.8×10.6 cm. A biopsy showed no definitive evidence of PTLD at that time, and EBV-PCR was undetectable.

**Figure 3:**
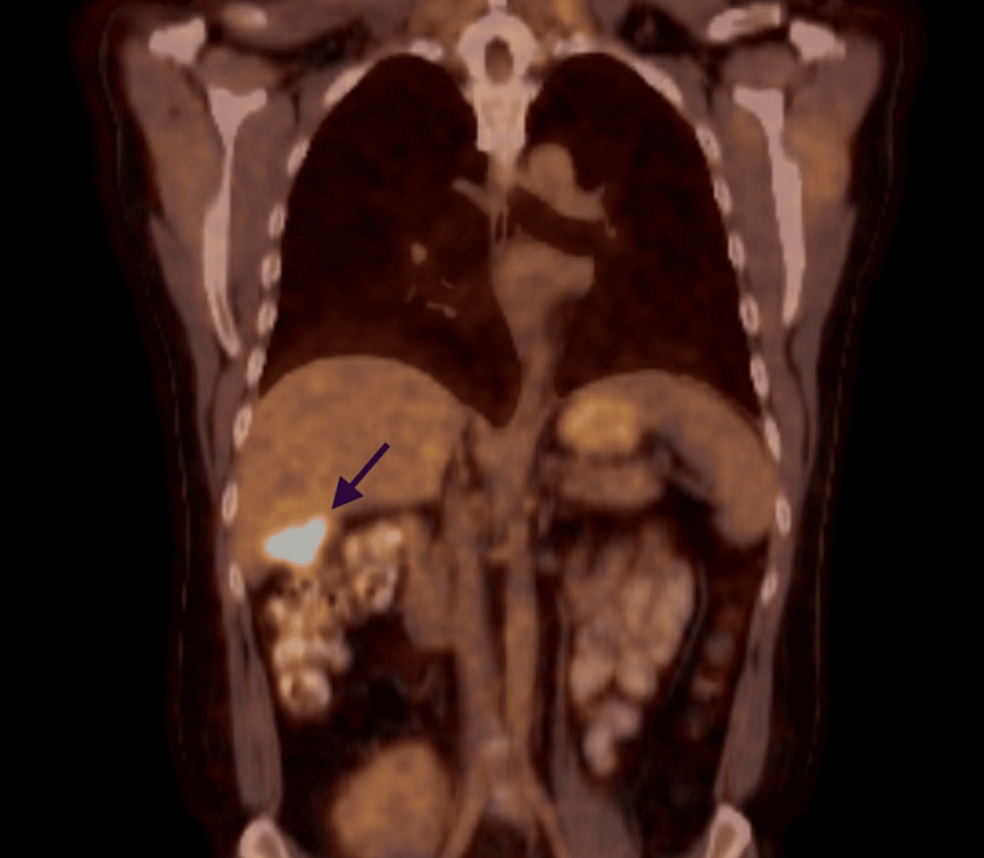
Increased uptake after two more doses of rituximab Arrow shows the increased uptake on repeat positron emission tomography-computer tomography (PET-CT) after two doses of rituximab.

Because of this, the patient received rituximab twice more as part of the Pola-R-CHP regimen, then another PET-CT scan two months later (Figure 3). This PET-CT scan showed progression of the same lesion with increased metabolic activity and size change to 9.0×6.3×7.9 cm.

The subsequent biopsy, for which eight samples were taken, resulted in necrosis with no viable lymphoma. The EBV-PCR continues to be undetectable at this time. The patient continues to be monitored with laboratory studies and a PET-CT surveillance scan every three months, with a plan to increase surveillance time in between scans depending on the stability of the next scan (due next month).

## Discussion

EBV-positive PTLD has distinct cytogenetics from EBV-negative PTLD [5]. For example, 9p21 is a chromosomal abnormality associated with EBV-positive PTLD only; this abnormality causes changes in cyclin-dependent kinase inhibitor expression and upregulation of the immune checkpoint receptor ligand programmed cell death ligand 2 (PD-L2) [6]. Another distinction is that early-onset PTLD is often associated with EBV-positive PTLD [7]. Regardless, although EBV status has been proven to guide therapy and response to therapy, neither EBV viral load nor EBV-positivity has been correlated to improved prognosis as yet.

Initial management of all patients with PTLD usually includes gradual RIS as early lesions can improve or resolve with this [8]. If refractory to RIS, rituximab, the anti-CD20 monoclonal antibody often used in the treatment of other CD20-positive lymphomas, is used as a single agent vs rituximab and combination chemotherapy. On one hand, rituximab as a single agent could be favorable with the plan to add a chemotherapeutic regimen if there is a lack of response. Twenty percent of patients with PTLD achieve complete response with single-agent rituximab [9], and side effects can be more tolerable. On the other hand, rituximab plus combination chemotherapy has been proven to reduce the risk of impairment of the transplanted organ, particularly in patients with renal transplants [10]. There is no standardized chemotherapy regimen chosen to pair with rituximab; however, R-CHOP is the most commonly prescribed treatment plan. With rituximab and chemotherapy, complete response is increased to 65% of patients [11].

In this patient, using the "POLARIX" trial as precedent, we had reason to treat with polatuzumab in his refractory PTLD [2], since the Pola-R-CHP regimen progression-free survival (PFS) was 76.7% vs 70.2% for RCHOP [3]. In particular, since this patient had a high viral EBV load and the associated ABC subtype (non-GCB) did better (83.9% vs 58.5% PFS) [3] in the trial, we were more inclined to be optimistic about his treatment. The patient tolerated the polatuzumab well. At this point, limitations include the follow-up time frame, as seeing the patient’s five-year survival rate would add value to this case.

## Conclusions

Due to this patient’s results on follow-up imaging and biopsies, there is reason to believe patients like him can benefit from the addition of polatuzumab if tolerated. As of now, the patient has completed six cycles of Pola-RCHP and two additional doses of rituximab. As we continue to follow his response to treatment, this case illustrates the potential for a new uniform treatment regimen in the setting of EBV+PTLD with a possibility for lower rates of the risk of disease progression, relapse, or death than the current standard of care.
